# Data on the pre-MDA and post MDA interventions for *Schistosoma mansoni* and *Schistosoma haematobium* in a co-endemic focus in Uganda: 1951–2011

**DOI:** 10.1016/j.dib.2018.08.200

**Published:** 2018-09-06

**Authors:** M. Adriko, B. Tinkitina, E.M. Tukahebwa, C.J. Standley, J.R. Stothard, N.B. Kabatereine

**Affiliations:** a Vector Control Division, Ministry of Health, P.O. Box 1661, Plot 15 Bombo Road, Kampala, Uganda; b Uganda Institute of Allied Health & Management Science (UIAHMS), School of Medical Entomology and Parasitology, P.O. Box 1661, Kampala, Uganda; c Ecology & Evolutionary Biology, Princeton University, Princeton, NJ 08544, USA; d Parasitology Department, Liverpool School of Tropical Medicine, Liverpool L3 5QA, UK; e Schistosomiasis Control Initiative, Department of Infectious Disease Epidemiology, Imperial College London, St Mary׳s Campus, Norfolk Place, London W2 1PG, UK

## Abstract

The dataset for this article contains Urinary and Intestinal Schistosomiasis from Lango region, northern Uganda which is the only known co-endemic region for *S.mansoni* and *S.haematobium*. Reported in the data, is the retrospective data review for historical information before interventions were implemented before 2003 and after interventions were implemented in 2003 by the national control program. In 2007 and 2011, parasitological surveys were conducted in the region to validate Schistosomiasis trends following World Health Organization (WHO) guidelines for surveys. In addition, malacological surveys were undertaken in 2007 to assess local transmission potential. The dataset can provide an insight into the health implications of Schistosomiasis control in co-endemic focus in Uganda, “*The epidemiology of schistosomiasis in Lango region Uganda 60 years after Schwetz 1951: Can schistosomiasis be eliminated through mass drug administration without other supportive control measures?*” (Adriko et al., 2018) [10].

**Specifications table**

**Table:** 

**Subject area**	*Neglected Tropical Diseases*
**More specific subject area**	*Schistosoma mansoni* and *Schistosoma haematobium in co-endemic focus in Uganda*
**Type of data**	*Tables and figures*
**How data was acquired**	*Field surveys involving collection and examination of stool and urine samples from school age children and adults*
**Data format**	*Raw and analyzed*
**Experimental factors**	*The above parameters in the abstract were analyzed according to WHO guidelines*
**Experimental features**	*Stool and Urine samples were analyzed according to WHO guidelines*[1]
**Data source location**	*Kampala, Uganda Latitude & Longitude for collected data are presented in this data article*
**Data accessibility**	*All data are within this article.*
**Related research article**	[10] Adriko, M., et al., *The epidemiology of schistosomiasis in Lango region Uganda 60 years after Schwetz 1951: Can schistosomiasis be eliminated through mass drug administration without other supportive control measures?* Acta Trop, 2018. **185**: p. 412–418.

**Value of the data**

• The dataset can be helpful to the concerned authorities and policy makers in designing interventions given the only region with co-endemic focus of the two disease species.
• The findings can be used by other researchers who wished to establish more insights into why the only region with co-endemic focuses for *S.mansoni* and *S.haematobium* in Uganda.
• The data can be used by the districts to validate health facility based detections.

## 1 Data

The data contains retrospective data review from studies [2], [3] and parasitological examination of urine samples for *S.haematobium* and stool samples for *S.mansoni* in 2007 and 2011 respectively. The datasets were collected from the Lango region of northern Uganda. Please See Table 1, Table 2, Table 3, Table 4, Table 5.

**Table 1:** Showing S. mansoni prevalence in Lango region, 2007.

**Site**	**Sample size**	**% Prevalence (95% CI)**
Abari Primary School	18	0.00 (0.00–18.53)
Abarolam community	29	3.45 (0.09–17.76)
Abilonino Primary School	20	0.00 (0.00–16.84)
Aceno Primary School	14	0.00 (0.00–23.16)
Agogoro Community	23	17.39 (4.95–38.78)
Aleka Primary School	16	56.25 (29.88–80.25)
Alenga Primary School	13	0.00 (0.00–24.71)
Alerwang Primary School	20	0.00 (0.00–16.84)
Aloi Community	30	40.00 (22.66–59.40)
Amuda Community	27	0.00 (0.00–12.77)
Aninolal Primary School	14	0.00 (0.00–23.16)
Apire Primary School	15	0.00 (0.00–21.80)
Atar Primary School	18	0.00 (0.00–18.53)
Atar Community	54	0.00 (0.00–6.60)
Atigolwok Primary School	28	0.00 (0.00–12.34)
Awala Primary School	29	10.34 (2.19–27.35)
Awila Primary School	12	8.33 (0.21–38.48)
Baradilo Primary School	19	0.00 (0.00–17.65)
Ebule Community	27	3.70 (0.09–18.97)
Loro Primary School	19	5.26 (0.13–26.03)
Odokogweno Community	29	0.00 (0.00–11.94)
Okole Primary School	21	4.76 (0.12–23.83)
Omer Primary School	15	0.00 (0.00–21.80)
Ongica Primary School	15	0.00 (0.00–21.80)
Teboke Primary School	20	0.00 (0.00–16.84)
Wansolo Primary School	55	69.09 (55.19–80.86)
Wigweng Primary School	14	0.00 (0.00–23.16)
**Total**	**627**	**11.32 (8.95–14.07)**

**Table 2:** Showing *S.haematobium* prevalence, by Hemastix result - 2011.

**Site**	**Sample Size**	**Trace = positive (% Prev.) (95% CI)**	**Trace = negative (% Prev.)(95% CI)**
Abako Com	61	0.00 (0.00–5.87)	0.00 (0.00–5.87)
Aber P/S	63	0.00 (0.00–5.69)	0.00 (0.00–5.69)
Abilonono Com	67	19.40 (10.76–30.89)	5.97 (1.65–14.59)
Abilonyero Com	57	3.51 (0.43–12.11)	3.51 (0.43–12.11)
Abwal-A Com	56	17.86 (8.91–30.40)	8.93 (2.96–19.62)
Acandyang Com	62	20.97 (11.66–33.18)	11.29 (4.66–21.89)
Adyanglim P/S	61	0.00 (0.00–5.87)	0.00 (0.00–5.87)
Agweng P/S	60	6.67 (1.85–16.20)	6.67 (0.00–5.87)
Akia P/S	62	8.06 (2.67–17.83)	8.06 (2.67–17.83)
Aleka P/S	69	1.45 (0.04–7.81)	0.00 (0.00–5.21)
Alenga P/S	63	12.70 (5.65–23.50)	3.17 (0.39–11.00)
Anget P/S	63	14.29 (6.75–25.39)	0.00 (0.00–5.69)
Apoi P/S	63	1.59 (0.04–8.53)	1.59 (0.04–8.53)
Atar P/S	64	7.81 (2.59–17.30)	4.69 (0.98–13.09)
Atigolwok P/S	65	27.69 (17.31–40.19)	10.77 (4.44–20.94)
Atoma Com	64	40.63 (28.51–53.63)	14.06 (6.64–25.02)
Awali P/S	64	4.69 (0.98–13.09)	4.69 (0.98–13.09)
Ayer P/S	62	24.19 (14.22–36.74)	24.19 (14.22–36.74)
Baraliro Com	62	3.23 (0.39–11.17)	3.23 (14.22–36.74)
Barocok P/S	59	3.39 (0.41–11.71)	3.39 (0.41–11.71)
Ebule P/S	66	4.55 (0.95–12.71)	4.55 (0.95–12.71)
Fatima Aloi P/S	66	3.03 (0.37–10.52)	3.03 (0.37–10.52)
Malika P/S	61	0.00 (0.00–5.87)	0.00 (0.00–5.87)
Obangangeo P/S	58	3.45 (0.42–11.91)	3.45 (0.42–11.91)
Ogogoro P/S	60	0.00 (0.00–5.96)	0.00 (0.00–5.96)
Ojul P/S	64	0.00 (0.00–5.60)	0.00 (0.00–5.60)
Olarokwon Com	61	0.00 (0.00–5.87)	0.00 (0.00–5.60)
Teboke P/S	65	32.31 (21.23–45.05)	9.23 (3.46–19.02)
Wansolo P/S	63	1.59 (0.04–8.53)	1.59 (0.04–8.53)
Wigua P/S	63	19.05 (10.25–30.91)	0.00 (0.00–5.96)
**Total**	**1874**	**9.50 (8.21–10.92)**	**3.74 (2.92–4.70)**

**Table 3:** Direct comparison of S.haematobium prevalence in sites surveyed both in 2007 and 2011, by Hemastix.

	**2007**		**2011**		
**Site**	**Sample size**	**Prevalence(95% CI)**	**Sample size**	**Prevalence(95% CI)**	**Trace = negative (% Prevalence)**
Abilonono**	20	0.00 (0.00–16.84)	67	19.40 (10.76–30.89)	5.97 (1.65–14.59)
Abilonono_2**	20	0.00 (0.00–16.84)			
Acandyang_A_com*	116	0.00 (0.00–3.13)	62	20.97 (11.66–33.18)	11.29 (4.66–21.89)
Acandyang_A2_com*	60	0.00 (0.00–5.96)			
Aleka	30	0.00 (0.00–11.57)	69	1.45 (0.04–7.81)	0.00 (0.00–5.21)
Alenga	30	0.00 (0.00–11.57)	63	12.70 (5.65–23.50)	3.17 (0.39–11.00)
Atigolwok	31	12.90 (3.63–29.83)	65	27.69 (17.31–40.19)	10.77 (4.44–20.94)
Atigolwok_com*	120	1.67 (0.20–5.89)			
Awali_com*	90	0.00 (0.00–4.02)	64	4.69 (0.98–13.09)	4.69 (0.98–13.09)
Barodilo**	20	90.00 (68.30–98.77)	62	3.23 (0.39–11.17)	3.23 (14.22–36.74)
Ebule_com*	120	0.00 (0.00–3.03)	66	4.55 (0.95–12.71)	4.55 (0.95–12.71)
Ogogoro_com*	118	0.00 (0.00–3.08)	60	0.00 (0.00–5.96)	0.00 (0.00–5.96)
Teboke	20	0.00 (0.00–16.84)	65	32.31 (21.23–45.05)	9.23 (3.46–19.02)
Teboke_2	20	0.00 (0.00–16.84)			
Wansola_com*	120	0.00 (0.00–3.03)	63	1.59 (0.04–8.53)	1.59 (0.04–8.53)
TOTAL	955	2.51 (1.61–3.72)

**Table 4:** Pre-MDA and Post-MDA Schistosomiasis Control in Lango region. The data Presented here shows the trends of co-endemic occurrence of *Schistosoma mansoni (S.m)* and *Schistosoma haematobium (S.h)* Pre-intervention (Mass Drug Administration, MDA) and Post-MDA.

**Data period**	**Year**	**Data Source**	**Current District**	**Survey District**	**School/Community**	**Lat**	**Long**	**Methods**	***% S.haem***	**Methods**	***% S.man***
Post-MDA	1992	[10]	Alebtong	Lira	Aloi school	2.51778	33.29500	Filtration	0.0	Kato Katz	82.0
Post-MDA	1992	[10]	Alebtong	Lira	Awali school	2.42295	33.07030	Filtration	0.0	Kato Katz	67.0
Post-MDA	1992	[10]	Alebtong	Lira	Namasale	1.51066	32.61987	Filtration	0.0	Kato Katz	38.0
Post-MDA	1992	[10]	Alebtong	Lira	Ogogoro school	2.21972	33.26806	Filtration	0.0	Kato Katz	63.0
Post-MDA	1992	[10]	Amolator	Lira	Aputi	1.83052	32.87519	Filtration	0.0	Kato Katz	42.0
Post-MDA	2007	[10]	Alebtong	Lira	Aloi school	2.51778	33.29500	Filtration	0.0	Kato Katz	33.3
Post-MDA	2007	[10]	Alebtong	Lira	Awali school	2.46167	33.29972	Filtration	0.0	Kato Katz	10.3
Post-MDA	2007	[10]	Alebtong	Lira	Ogogoro school	2.21972	33.26806	Filtration	0.0	Kato Katz	14.8
Post-MDA	2007	[10]	Apac	Apac	Akokoro school	1.78000	32.56333	Filtration	0.0	Kato Katz	0.0
Post-MDA	2007	[10]	Apac	Apac	Alenga school	1.10361	32.40750	Filtration	0.0	Kato Katz	0.0
Post-MDA	2007	[10]	Apac	Apac	Alerwang school	2.16833	32.55639	Filtration	0.0	Kato Katz	0.0
Post-MDA	2007	[10]	Apac	Apac	Aninolal school	2.25806	32.63278	Filtration	0.0	Kato Katz	0.0
Post-MDA	2007	[10]	Apac	Apac	Apire school	2.01861	32.91500	Filtration	0.0	Kato Katz	0.0
Post-MDA	2007	[10]	Apac	Apac	Atar community	2.13528	32.66056	Filtration	0.9	Kato Katz	0.0
Post-MDA	2007	[10]	Apac	Apac	Atar school	2.04056	32.58972	Filtration	7.5	Kato Katz	0.0
Post-MDA	2007	[10]	Apac	Apac	Atigolwo school	2.34361	32.65333	Filtration	10.3	Kato Katz	0.0
Post-MDA	2007	[10]	Apac	Apac	Atogolwo com	2.34389	32.70361	Filtration	1.7	Kato Katz	0.0
Post-MDA	2007	[10]	Apac	Apac	Awila school	1.03583	32.48111	Filtration	0.0	Kato Katz	0.0
Post-MDA	2007	[10]	Apac	Apac	Barodilo school	2.21222	32.73222	Filtration	0.0	Kato Katz	0.0
Post-MDA	2007	[10]	Apac	Apac	Chegere school	2.23667	32.62917	Filtration	0.0	Kato Katz	0.0
Post-MDA	2007	[10]	Apac	Apac	Ikwera school	2.12944	32.94028	Filtration	0.0	Kato Katz	0.0
Post-MDA	2007	[10]	Apac	Apac	Kwibale school	1.69722	32.33389	Filtration	0.0	Kato Katz	0.0
Post-MDA	2007	[10]	Apac	Apac	Okutoagwe school	2.26333	32.63278	Filtration	0.0	Kato Katz	0.0
Post-MDA	2007	[10]	Apac	Apac	Omer school	2.04778	32.79028	Filtration	0.0	Kato Katz	0.0
Post-MDA	2007	[10]	Apac	Apac	Ongica school	2.34083	32.86000	Filtration	0.0	Kato Katz	0.0
Post-MDA	2007	[10]	Apac	Apac	Teboke school	2.45778	32.67389	Filtration	0.0	Kato Katz	0.0
Post-MDA	2007	[10]	Apac	Apac	Wansolo com	1.75528	32.72556	Filtration	0.0	Kato Katz	58.6
Post-MDA	2007	[10]	Dokolo	Lira	Amuda school	1.23861	33.02083	Filtration	0.0	Kato Katz	0.0
Post-MDA	2007	[10]	Kole	Apac	Abari school	2.30583	32.70583	Filtration	0.0	Kato Katz	0.0
Post-MDA	2007	[10]	Kole	Apac	Abelonino school	2.39500	32.85667	Filtration	0.0	Kato Katz	0.0
Post-MDA	2007	[10]	Kole	Apac	Damatira school	2.32417	32.80528	Filtration	0.0	Kato Katz	0.0
Post-MDA	2007	[10]	Kole	Apac	Okole school	2.43222	32.66056	Filtration	0.0	Kato Katz	4.8
Post-MDA	2007	[10]	Lira	Lira	Abarolam school	1.02028	33.16917	Filtration	0.0	Kato Katz	3.4
Post-MDA	2007	[10]	Lira	Lira	Ebule school	2.15333	33.55306	Filtration	0.0	Kato Katz	3.7
Post-MDA	2007	[10]	Lira	Lira	Odekogweno	1.03972	33.27778	Filtration	0.0	Kato Katz	0.0
Post-MDA	2007	[10]	Oyam	Oyam	Acaba school	2.60694	32.61444	Filtration	0.0	Kato Katz	0.0
Post-MDA	2007	[10]	Oyam	Oyam	Aceno school	2.46944	32.65583	Filtration	0.0	Kato Katz	0.0
Post-MDA	2007	[10]	Oyam	Oyam	Ader school	2.54944	32.91528	Filtration	0.0	Kato Katz	0.0
Post-MDA	2007	[10]	Oyam	Oyam	Aleka school	2.72806	32.85417	Filtration	0.0	Kato Katz	46.7
Post-MDA	2007	[10]	Oyam	Oyam	Anget school	2.75500	32.81278	Filtration	3.3	Kato Katz	23.1
Post-MDA	2007	[10]	Oyam	Oyam	Loro school	2.23861	32.53611	Filtration	0.0	Kato Katz	5.0
Post-MDA	2007	[10]	Oyam	Oyam	Obot school	2.46972	32.60389	Filtration	0.0	Kato Katz	0.0
Post-MDA	2007	[10]	Oyam	Oyam	Onegwok	2.64444	32.69667	Filtration	0.0	Kato Katz	0.0
Post-MDA	2007	[10]	Oyam	Oyam	Wigweng school	2.45667	32.70583	Filtration	0.0	Kato Katz	0.0
Post-MDA	2008	[10]	Alebtong	Lira	Abako Com	2.14602	33.22521	Filtration	0.0	Kato Katz	20.3
Post-MDA	2008	[10]	Alebtong	Lira	Ogogoro P/S	2.18874	33.20177	Filtration	0.0	Kato Katz	13.4
Post-MDA	2008	[10]	Alebtong	Lira	Ojul P/S	2.12264	33.20377	Filtration	0.0	Kato Katz	1.7
Post-MDA	2008	[10]	Amolatar	Amolatar	Muntu P/S	1.58197	32.89720	Filtration	0.0	Kato Katz	2.9
Post-MDA	2008	[10]	Amolatar	Amolatar	Namasale P/S	1.51066	32.61987	Filtration	0.0	Kato Katz	1.6
Post-MDA	2008	[10]	Amolatar	Amolatar	Opir P/S	1.55203	32.82683	Filtration	0.0	Kato Katz	2.5
Post-MDA	2008	[10]	Oyam	Oyam	Atur Com	2.13525	32.33604	Filtration	0.0	Kato Katz	9.6
Post-MDA	2008	[10]	Oyam	Oyam	Nora P/S	2.29298	32.26281	Filtration	0.0	Kato Katz	0.4
Post-MDA	2009	[10]	Alebtong	Lira	Ogogoro p/s	2.18874	33.20177	Filtration	0.0	Kato Katz	5.8
Post-MDA	2009	[10]	Alebtong	Lira	Ojul p/s	2.12264	33.20377	Filtration	0.0	Kato Katz	2.0
Post-MDA	2009	[10]	Amolator	Amolator	Muntu p/s	1.58197	32.89720	Filtration	0.0	Kato Katz	2.1
Post-MDA	2009	[10]	Amolator	Amolator	Opir p/s	1.55203	32.82683	Filtration	0.0	Kato Katz	3.8
Post-MDA	2011	[10]	Alebtong	Alebtong	Abako Com	2.14602	33.22521	Filtration	0.0	Kato Katz	18.0
Post-MDA	2011	[10]	Alebtong	Alebtong	Adyanglim	2.10130	33.21291	Filtration	0.0	Kato Katz	9.8
Post-MDA	2011	[10]	Alebtong	Alebtong	Awali	2.42295	33.07030	Filtration	0.0	Kato Katz	17.2
Post-MDA	2011	[10]	Alebtong	Alebtong	Ebule	2.15339	33.36017	Filtration	0.0	Kato Katz	3.0
Post-MDA	2011	[10]	Alebtong	Alebtong	Fatima Aloi Demo	2.26912	33.14071	Filtration	0.0	Kato Katz	29.7
Post-MDA	2011	[10]	Alebtong	Alebtong	Obangangeo	2.18572	33.36504	Filtration	0.0	Kato Katz	0.0
Post-MDA	2011	[10]	Alebtong	Alebtong	Ogogoro	2.18874	33.20177	Filtration	0.0	Kato Katz	11.7
Post-MDA	2011	[10]	Alebtong	Alebtong	Ojul	2.12264	33.20377	Filtration	0.0	Kato Katz	6.3
Post-MDA	2011	[10]	Apac	Apac	Abwal A com	2.08301	32.55774	Filtration	0.0	Kato Katz	0.0
Post-MDA	2011	[10]	Apac	Apac	Acandyang com	2.00903	32.60387	Filtration	11.3	Kato Katz	1.6
Post-MDA	2011	[10]	Apac	Apac	Alenga	1.84964	32.35359	Filtration	0.0	Kato Katz	1.6
Post-MDA	2011	[10]	Apac	Apac	Apoi	1.73001	32.46858	Filtration	0.0	Kato Katz	1.6
Post-MDA	2011	[10]	Apac	Apac	Atar	2.04032	32.59378	Filtration	1.6	Kato Katz	0.0
Post-MDA	2011		Apac	Apac	Atigolwok	2.08349	32.55926	Filtration	0.0	Kato Katz	0.0
Post-MDA	2011	[10]	Apac	Apac	Atoma Com	1.81005	32.75776	Filtration	0.0	Kato Katz	23.0
Post-MDA	2011	[10]	Apac	Apac	Teboke	2.19976	32.58875	Filtration	0.0	Kato Katz	0.0
Post-MDA	2011	[10]	Apac	Apac	Wansolo	1.67725	32.50212	Filtration	0.0	Kato Katz	49.2
Post-MDA	2011	[10]	Kole	Kole	Abilonono Com	2.22691	32.64050	Filtration	0.0	Kato Katz	1.5
Post-MDA	2011	[10]	Kole	Kole	Ayer	2.29128	32.71657	Filtration	0.0	Kato Katz	1.6
Post-MDA	2011	[10]	Kole	Kole	Wigua	2.36085	32.67409	Filtration	0.0	Kato Katz	1.6
Post-MDA	2011	[10]	Lira	Lira	Agweng	2.49592	32.93468	Filtration	0.0	Kato Katz	42.9
Post-MDA	2011	[10]	Lira	Lira	Akia	2.25183	32.94784	Filtration	0.0	Kato Katz	3.9
Post-MDA	2011	[10]	Otuke	Otuke	Abilonyero com	2.40515	33.23411	Filtration	0.0	Kato Katz	0.0
Post-MDA	2011	[10]	Otuke	Otuke	Baraliro com	2.47074	33.17848	Filtration	0.0	Kato Katz	3.2
Post-MDA	2011	[10]	Otuke	Otuke	Barocok	2.50718	33.10303	Filtration	0.0	Kato Katz	5.1
Post-MDA	2011	[10]	Otuke	Otuke	Malika	2.43519	33.25354	Filtration	0.0	Kato Katz	11.5
Post-MDA	2011	[10]	Otuke	Otuke	Olarokwon com	2.51360	33.26091	Filtration	0.0	Kato Katz	0.0
Post-MDA	2011	[10]	Oyam	Oyam	Aber	2.20114	32.34769	Filtration	0.0	Kato Katz	1.6
Post-MDA	2011	[10]	Oyam	Oyam	Aleka	2.56069	32.75598	Filtration	0.0	Kato Katz	43.3
Post-MDA	2011	[10]	Oyam	Oyam	Anget	2.57830	32.78435	Filtration	0.0	Kato Katz	32.3
Pre-MDA	1951	[2]		Oyam	Aloro			Direct Micro	28.6	Direct Micro	0.0
Pre-MDA	1951	[2]		Apac	Ayer			Direct Micro	39.3	Direct Micro	0.0
Pre-MDA	1951	[2]		Apac	Aboki			Direct Micro	33.3	Direct Micro	0.0
Pre-MDA	1951	[2]		Apac	Nyunbuke Catholic			Direct Micro	0.0	Direct Micro	0.0
Pre-MDA	1951	[2]		Apac	Nyunbuke Protestant			Direct Micro	0.0	Direct Micro	0.0
Pre-MDA	1951	[2]		Apac	Aber Protestant			Direct Micro	20.0	Direct Micro	0.0
Pre-MDA	1951	[2]		Apac	Adyegi			Direct Micro	0.0	Direct Micro	0.0
Pre-MDA	1951	[2]		Apac	Ibuje-Alenga			Direct Micro	27.3	Direct Micro	0.0
Pre-MDA	1951	[2]		Apac	Akokoro			Direct Micro	0.0	Direct Micro	0.0
Pre-MDA	1951	[2]		Apac	Nyalu Village			Direct Micro	0.0	Direct Micro	0.0
Pre-MDA	1967	[2]		Kole	Abilonino			Filtration	51.6	Formal- ether	0.0
Pre-MDA	1967	[3]		Apac	Abiya			Filtration	0.0	Formal- ether	3.6
Pre-MDA	1967	[3]		Apac	Aduku			Filtration	0.0	Formal- ether	0.0
Pre-MDA	1967	[3]		Lira	Akia (Lira)			Filtration	0.0	Formal- ether	10.3
Pre-MDA	1967	[3]		Alebtong	Aloi			Filtration	0.0	Formal- ether	0.0
Pre-MDA	1967	[3]		Lira	Atura			Filtration	0.0	Formal- ether	0.0
Pre-MDA	1967	[3]		Amolator	Muntu			Filtration	0.0	Formal- ether	0.0
Pre-MDA	1967	[3]		Otuke	Paranga			Filtration	0.0	Formal- ether	53.3
Pre-MDA	1967	[3]		Oyam	Teboke			Filtration	0.0	Formal- ether	0.0

**Table 5:** Snail data model results.

**Snail species**	**Dependent variable**	**Factor (baseline; category)**	**Odds ratio (95% CI)**	**p- value**
*Bi. sudanica*	Presence/Absence	altitude (meters); continuous (+ 1)	0.95 (0.89–1.01)	0.073
		Temperature (C); Continuous (+ 0.1)	0.61 (0.41–0.91)	0.017
*Bi. pfeifferi*	Abundance	pH; continuous (+ 0.1)	564.45* (5.50–5.794)	0.010
*Bu. forskalii*	Presence/Absence	altitude (meters); continuous (+ 1)	0.96 (0.93–0.99)	0.019
	Abundance	altitude (meters); continuous (+ 1)	0.93 (0.87–0.99)	0.021
*Bu. tropicus*	Presence/Absence	altitude (meters); continuous (+ 1)	1.02 (1.00–1.04)	0.074

* Note pH is on a logarithmic scale, and so an odds ratio of 10 corresponds to an increase of 1 on the pH scale, an increase of 100 corresponds to 2 pH points, etc. No factors were significant in predicting the presence/absence or abundance of snails infected with non-human cercariae.

## 2 Experimental designs, methods and materials

This study related to the data was carried out in the former Lango district previously described by [2]. About 20 ml of urine were collected and tested for the presence of microhaematuria using reagent strips (Hemastix©, Bayer, Germany) and recorded following grading [4]. For confirmation of the infection, a syringe filtration method [5] and examined for schistosome eggs [1] while stool samples for S.mansoni infections were processed using Kato-Katz double thick smears [6] using a 41.7 mg template and duplicate smears examined under a microscope according to WHO guidelines [1]. Snail surveys were conducted in 2007 in the vicinity of each school surveyed for Bulinus and Biomphalaria snail species following guidelines [7] and identified using field keys [8] and [9]. The following datasets are presented.

### 2.1 2007 data

The Table 1 below shows the data on the generalized linear model (GLM) looking at factors influencing binomial prevalence of *S. haematobium* infection (as diagnosed by Hemastix), with inclusion of age, sex and knowledge of bilharzia as explanatory variables.

### 2.2 2011 data

The 2011 data presented in Table 2 shows *S. mansoni* infection, the relationships with sex and age amongst those surveyed.

### 2.3 Direct comparison of *S.haematobium* prevalence in sites surveyed both in 2007 and 2011, by Hemastix

In several cases, multiple surveys had been conducted in the same region in 2007 whereas only a single survey was carried out in 2011. In some cases, the survey in 2007 took place in the community whereas the follow-up in 2011 took place in the local primary school; these cases are marked with “*”. The inverse cases, where the initial survey took place in a primary school and the follow-up in the community, are marked with “**”. Urine syringe filtration was only carried out in 2011 (Table 3).

### 2.4 Snail data model

All models were multivariate, including altitude, temperature, pH, conductivity and dissolved oxygen as covariates. Presence/absence models were estimated using a generalized linear model (glm) whereas abundance mo dels were estimated using a linear model (lm) with only factors that had a *p*-value less than 0.1 included(at the 95% confidence level) (Table 5).

## References

[1] WHO (2002). Prevention and Control of Schistosomiasis and Soil-transmitted Helminthiasis - Report of a WHO Expert Committee.

[2] Schwetz J. (1951). On vesical bilharzia in the Lango district (Uganda). Trans. R. Soc. Trop. Med. Hyg..

[3] Bradley D.J., Sturrock R.F., Williams P.N. (1967). The circumstantial epidemiology of Schistosoma haematobium in Lango district, Uganda. East Afr. Med J.

[4] Wilkins H.A., Goll P., Marshall T.F., Moore P. (1979). The significance of proteinuria and haematuria in Schistosoma haematobium infection. Trans. R Soc. Trop. Med Hyg..

[5] Peters P.A., Mahmoud A.A., Warren K.S., Ouma J.H., Siongok T.K. (1976). Field studies of a rapid, accurate means of quantifying Schistosoma haematobium eggs in urine samples. Bull. World Health Organ.

[6] Katz N., Chaves A., Pellegrino J. (1972). A simple device for quantitative stool thick-smear technique in Schistosomiasis mansoni. Rev. Inst. De. Med. Trop. De. Sao Paulo.

[7] Madsen (1985). Ecology and Control of African Freshwater Pulmonate Snails; Part 1: Life Cycle and Methodology.

[8] Kristensen T.K. (1987). A Field Guide to African Fresh Water Snails.

[9] Frandsen F., Christensen N.Ø. (1984). An introductory guide to the identification of cercariae from Afican freshwater snails with special reference to cercariae of trematode species of medical and veterinary importance. Acta Trop..

[10] Adriko M., Tinkitina B., Tukahebw E.M., Standley C.J., Stothard J.R. (2018). The epidemiology of schistosomiasis in Lango region Uganda 60 years after Schwetz 1951: can schistosomiasis be eliminated through mass drug administration without other supportive control measures?. Acta Trop..

