# Supplementary material for: Data on the pre-MDA and post MDA interventions for Schistosoma mansoni and Schistosoma haematobium in a co-endemic focus in Uganda: 1951–2011
Source: Data Brief. 2018 Sep 6;20:991–8. doi: 10.1016/j.dib.2018.08.200 (PMC6138995; doi:10.1016/j.dib.2018.08.200)
Supplement: Supplementary file 1 — Supplementary material. [file mmc1.docx]

**Confirmation of contribution by authors and copyright agreement**

11^th^ July 2018.

Dear Prof Brattig,

We would like to submit our article for your consideration for publication in Acta Tropica.

Our manuscript is entitled **“Data on the Pre-MDA and Post MDA interventions for *Schistosoma mansoni* and *Schistosoma haematobium* in a Co-endemic focus in Uganda: 1951-2011**”

We feel that this data would bee of interest to those who work on the elimination of Schistosomiasis in endemic countries, especially in Sub-Saharan Africa. This work is original and has not been submitted for publication elsewhere.

**Conflict of interest**

None of the authors have a conflict of interest.

Yours faithfully,

| Adriko Moses |  |
| --- | --- |
| Benjamin Tinkitina |  |
| Edridah M. Tukahebwa |  |
| Claire J. Standley |  |
| J. R. Stothard |  |
| Narcis B. Kabatereine |  |
